# A MIXED-METHODS STUDY OF NURSING HOME RESIDENTS’ EXPERIENCES OF RELATIONSHIPS AND DAY-TO-DAY SOCIAL INTERACTIONS

**DOI:** 10.1093/geroni/igz038.2045

**Published:** 2019-11-08

**Authors:** Allyson M Washburn, Susan Williams

**Affiliations:** 1 National University, La Jolla, California, United States; 2 National University, Stockton, California, United States

## Abstract

Nursing home residents with and without cognitive impairment (N=38) answered open-ended questions about their day-to-day social interactions and ongoing relationships with family and friends. One author (SW) completed a conventional content analysis of the transcripts and the other (AW), a phenomenological-hermeneutic analysis. Findings from these analyses were combined and examined further using data from measures of social cognition and staff ratings of social behavior. Participants’ social experiences appeared to be determined not only by long-established habits and preferences and length of nursing home stay but also by their cognitive status and social cognition competencies. A central theme was the importance of managing ongoing relationships and day-to-day interactions so as to reduce one’s own stress as well as the burden on others. This presentation details how findings from distinct analytic strategies were combined to characterize the researchers’ understanding of participants’ lives in their networks of care from their own perspective.

